# Supplementary material for: General, open-source vertex modeling in biological applications using Tissue Forge
Source: Sci Rep. 2023 Oct 19;13:17886. doi: 10.1038/s41598-023-45127-x (PMC10587242; doi:10.1038/s41598-023-45127-x)
Supplement: Supplementary file 1 — Supplementary Information. [file 41598_2023_45127_MOESM1_ESM.zip › Supplementary Information/Supplementary Texts.docx]

General, Open-Source Vertex Modeling in Biological Applications Using Tissue Forge

T.J. Sego^1*^, Tien Comlekoglu^2, 3^, Shayn M. Peirce^2^, Douglas W. Desimone^3^, James A. Glazier^4^

^1^ Department of Medicine, University of Florida, Gainesville, FL, USA

^2^ Department of Biomedical Engineering, University of Virginia, Charlottesville, VA, USA

^3^ Department of Cell Biology, University of Virginia, Charlottesville, VA, USA

^4^ Department of Intelligent Engineering and Biocomplexity Institute, Indiana University, Bloomington, IN, USA

^*^ timothy.sego@ufl.edu

Supplementary Text S1. Installing Tissue Forge.

The Tissue Forge GitHub repository and documentation are available at the following links,

- Tissue Forge repository: https://github.com/tissue-forge/tissue-forge
- Tissue Forge documentation: https://tissue-forge-documentation.readthedocs.io/en/latest
- Tissue Forge Python API documentation: https://tissue-forge-python-api-documentation.readthedocs.io/en/latest

Tissue Forge v0.1.0 installations are available for 64-bit Windows, Linux and MacOS via conda from the *tissue-forge* channel. Tissue Forge can be installed in a terminal with the *conda* command available using the following command,

*conda install –c conda-forge –c tissue-forge tissue-forge*

The Tissue Forge C, C++ and Python APIs are available in any conda environment in which the Tissue Forge is installed after activating the conda environment.

Supplementary Text S2. Actor Forms.

## Actors on surfaces

### Adhesion

For surfaces $\mathcal{S}_{1}$ and $\mathcal{S}_{2}$, adhesion between $\mathcal{S}_{1}$ and $\mathcal{S}_{2}$ is defined by the effective energy,

$$\mathcal{H}\left( \mathcal{S}_{1}, \mathcal{S}_{2} \right)=\lambda\left( \mathcal{S}_{1}, \mathcal{S}_{2} \right)L\left( \mathcal{S}_{1}, \mathcal{S}_{2} \right).$$

Here $\lambda\left( \mathcal{S}_{1}, \mathcal{S}_{2} \right)$ is a model parameter and $L\left( \mathcal{S}_{1}, \mathcal{S}_{2} \right)$ is the length of the edges connecting $\mathcal{S}_{1}$ and $\mathcal{S}_{2}$.

### Convex Polygon Constraint

If $\mathcal{V}_{k}$ with connected $\mathcal{V}_{k-1}$ and $\mathcal{V}_{k+1}$ makes a surface $\mathcal{S}$ non-convex, then the convex polygon constraint applies the force $f_{i}\left( \mathcal{V}_{k} \right)$ on $\mathcal{V}_{k}$,

$$f_{i}\left( \mathcal{V}_{k} \right)=\frac{\lambda M^{\mathcal{V}}\left( \mathcal{V}_{k} \right)}{\Delta t}\left( r_{i}\left( \mathcal{V}_{k-1} \right)-r_{i}\left( \mathcal{V}_{k} \right)+\frac{1}{\left\| l_{n} \right\|^{2}}\left( r_{j}\left( \mathcal{V}_{k} \right)-r_{j}\left( \mathcal{V}_{k-1} \right) \right)l_{j}l_{i} \right),$$

where $\lambda$ is a parameter, $M^{\mathcal{V}}\left( \mathcal{V}_{k} \right)$ is the drag of $\mathcal{V}_{k}$, $\Delta t$ is the simulation time step, and

$$l_{i}=\frac{r_{i}\left( \mathcal{V}_{k+1} \right)-r_{i}\left( \mathcal{V}_{k-1} \right)}{\left\| r_{i}\left( \mathcal{V}_{k+1} \right)-r_{i}\left( \mathcal{V}_{k-1} \right) \right\|}.$$

Test for convexity is implemented by measuring the area $A$ of the triangle formed by the surface centroid $C_{i}^{\mathcal{S}}\left( \mathcal{S} \right)$, $r_{i}\left( \mathcal{V}_{k-1} \right)$ and $r_{i}\left( \mathcal{V}_{k+1} \right)$, and comparing $A$ with the sum $A^{'}$ of the area of the triangles formed by the triplets $\left\{ r_{i}\left( \mathcal{V}_{k-1} \right), r_{i}\left( \mathcal{V}_{k} \right), r_{i}\left( \mathcal{V}_{k+1} \right) \right\}$, $\left\{ r_{i}\left( \mathcal{V}_{k} \right), r_{i}\left( \mathcal{V}_{k+1} \right), C_{i}^{\mathcal{S}}\left( \mathcal{S} \right) \right\}$ and $\left\{ r_{i}\left( \mathcal{V}_{k-1} \right), r_{i}\left( \mathcal{V}_{k} \right), C_{i}^{\mathcal{S}}\left( \mathcal{S} \right) \right\}$. If $A=A^{'}$, then $\mathcal{V}_{k}$ makes the surface non-convex.

### Edge Tension

Edge tension for surface $\mathcal{S}$ is defined by the effective energy,

$$\mathcal{H}\left( \mathcal{S} \right)=\lambda\left( \mathcal{S} \right)L^{n}\left( \mathcal{S} \right), n>0.$$

Here $\lambda\left( \mathcal{S} \right)$ and $n$ are model parameters and $L\left( \mathcal{S} \right)$ is the perimeter of $\mathcal{S}$.

### Flat Surface Constraint

The flat surface constraint applies a force $f_{i}\left( \mathcal{V} \right)$ to vertex $\mathcal{V}$ for every surface $\mathcal{S}$ that it defines,

$$f_{i}\left( \mathcal{V} \right)=\frac{\lambda M^{\mathcal{V}}\left( \mathcal{V} \right)}{\Delta t}\left( \left( C_{j}^{\mathcal{S}}\left( \mathcal{S} \right)-r_{j}\left( \mathcal{V} \right) \right)\eta_{j}^{\mathcal{S}}\left( \mathcal{S} \right)\eta_{i}^{\mathcal{S}}\left( \mathcal{S} \right) \right).$$

Here $\lambda$ is a parameter, $M^{\mathcal{V}}\left( \mathcal{V}_{k} \right)$ is the drag of $\mathcal{V}_{k}$, $\eta_{i}^{\mathcal{S}}\left( \mathcal{S} \right)$ is the unit normal of $\mathcal{S}$, and $\Delta t$ is the simulation time step.

### Normal Stress

Normal stress applied to a surface $\mathcal{S}$ with magnitude $F\left( \mathcal{S} \right)$ results in a force $f_{i}\left( \mathcal{V} \right)$ applied to every vertex $\mathcal{V}$ that defines the surface,

$$f_{i}\left( \mathcal{V} \right)=\frac{A\left( \mathcal{V; S} \right)}{A\left( \mathcal{S} \right)}F\left( \mathcal{S} \right)\eta_{i}^{\mathcal{S}}\left( \mathcal{S} \right).$$

Here $A\left( \mathcal{V; S} \right)$ is the area contribution of $\mathcal{V}$ to$\mathcal{S}$, $A\left( \mathcal{S} \right)$ is the area of $\mathcal{S}$, $F\left( \mathcal{S} \right)$ is the magnitude of the force, and $\eta_{i}^{\mathcal{S}}\left( \mathcal{S} \right)$ is the unit normal of $\mathcal{S}$.

### Perimeter Constraint

The perimeter constraint imposes that the perimeter of a surface $\mathcal{S}$ tends towards a value with the effective energy,

$$\mathcal{H}\left( \mathcal{S} \right)=\lambda\left( \mathcal{S} \right)\left( L\left( \mathcal{S} \right)-L^{o}\left( \mathcal{S} \right) \right)^{2}.$$

Here $\lambda$ is a parameter, $L\left( \mathcal{S} \right)$ is the perimeter of $\mathcal{S}$ and $L^{o}\left( \mathcal{S} \right)$ is the target perimeter of $\mathcal{S}$.

### Surface Area Constraint

The surface area constraint imposes that the area of a surface $\mathcal{S}$ tends towards a value with the effective energy,

$$\mathcal{H}\left( \mathcal{S} \right)=\lambda\left( \mathcal{S} \right)\left( A\left( \mathcal{S} \right)-A^{o}\left( \mathcal{S} \right) \right)^{2}.$$

Here $\lambda$ is a parameter, $A\left( \mathcal{S} \right)$ is the area of $\mathcal{S}$ and $A^{o}\left( \mathcal{S} \right)$ is the target area of $\mathcal{S}$.

### Surface Traction

Surface traction applied to a surface $\mathcal{S}$ with traction $\tau_{i}\left( \mathcal{S} \right)$ results in a force $f_{i}\left( \mathcal{V} \right)$ applied to every vertex $\mathcal{V}$ that defines the surface,

$$f_{i}\left( \mathcal{V} \right)=A\left( \mathcal{V; S} \right)\tau_{i}\left( \mathcal{S} \right).$$

Here $A\left( \mathcal{V; S} \right)$ is the area contribution of $\mathcal{V}$ to $\mathcal{S}$.

## Actors on bodies

### Adhesion

For bodies $\mathcal{B}_{1}$ and $\mathcal{B}_{2}$, adhesion between $\mathcal{B}_{1}$ and $\mathcal{B}_{2}$ is defined by the effective energy,

$$\mathcal{H}\left( \mathcal{B}_{1}, \mathcal{B}_{2} \right)=\lambda\left( \mathcal{B}_{1}, \mathcal{B}_{2} \right)A\left( \mathcal{B}_{1}, \mathcal{B}_{2} \right)$$

Here $\lambda\left( \mathcal{B}_{1}, \mathcal{B}_{2} \right)$ is a model parameter and $A\left( \mathcal{B}_{1}, \mathcal{B}_{2} \right)$ is the area of the surfaces connecting $\mathcal{B}_{1}$ and $\mathcal{B}_{2}$.

### Body Force

Body force applied to a body $\mathcal{B}$ with force $f_{i}^{\mathcal{B}}\left( \mathcal{B} \right)$ results in a force $f_{i}\left( \mathcal{V} \right)$ applied to every vertex $\mathcal{V}$ that defines the body,

$$f_{i}\left( \mathcal{V} \right)=\frac{V\left( \mathcal{V; B} \right)}{V\left( \mathcal{B} \right)}f_{i}^{\mathcal{B}}\left( \mathcal{B} \right).$$

Here $V\left( \mathcal{V; B} \right)$ is the volume contribution of $\mathcal{V}$ to $\mathcal{B}$ and $V\left( \mathcal{B} \right)$ is the volume of $\mathcal{B}$.

### Surface Area Constraint

The surface area constraint imposes that the surface area of a body $\mathcal{B}$ tends towards a value with the effective energy,

$$\mathcal{H}\left( \mathcal{B} \right)=\lambda\left( \mathcal{B} \right)\left( A\left( \mathcal{B} \right)-A^{o}\left( \mathcal{B} \right) \right)^{2}$$

Here $\lambda$ is a parameter, $A\left( \mathcal{B} \right)$ is the surface area of $\mathcal{B}$ and $A^{o}\left( \mathcal{B} \right)$ is the target surface area of $\mathcal{B}$.

### Volume Constraint

The volume constraint imposes that the volume of a body $\mathcal{B}$ tends towards a value with the effective energy,

$$\mathcal{H}\left( \mathcal{B} \right)=\lambda\left( \mathcal{B} \right)\left( V\left( \mathcal{B} \right)-V^{o}\left( \mathcal{B} \right) \right)^{2}$$

Here $\lambda$ is a parameter, $V\left( \mathcal{B} \right)$ is the volume of $\mathcal{B}$ and $V^{o}\left( \mathcal{B} \right)$ is the target volume of $\mathcal{B}$.
